# NF-κB p65 Subunit Is Modulated by Latent Transforming Growth Factor-β Binding Protein 2 (LTBP2) in Nasopharyngeal Carcinoma HONE1 and HK1 Cells

**DOI:** 10.1371/journal.pone.0127239

**Published:** 2015-05-14

**Authors:** Rebecca Kan, Wai Ho Shuen, Hong Lok Lung, Arthur Kwok Leung Cheung, Wei Dai, Dora Lai-Wan Kwong, Wai Tong Ng, Anne Wing Mui Lee, Chun Chung Yau, Roger Kai Cheong Ngan, Stewart Yuk Tung, Maria Li Lung

**Affiliations:** 1 Department of Clinical Oncology, Li Ka Shing Faculty of Medicine, University of Hong Kong, Hong Kong (SAR), PR China; 2 Center for Cancer Research, Li Ka Shing Faculty of Medicine, University of Hong Kong, Hong Kong (SAR), PR China; 3 Center for Nasopharyngeal Carcinoma Research, Li Ka Shing Faculty of Medicine, University of Hong Kong, Hong Kong (SAR), PR China; 4 Department of Clinical Oncology, Pamela Youde Nethersole Eastern Hospital, Hong Kong (SAR), PR China; 5 Department of Clinical Oncology, The University of Hong Kong—Shen Zhen Hospital, PR China; 6 Department of Oncology, Princess Margaret Hospital, Hong Kong (SAR), PR China; 7 Department of Clinical Oncology, Queen Elizabeth Hospital, Hong Kong (SAR), PR China; 8 Department of Clinical Oncology, Tuen Mun Hospital, Hong Kong (SAR), PR China; Gustave Roussy, FRANCE

## Abstract

NF-κB is a well-characterized transcription factor, widely known as a key player in tumor-derived inflammation and cancer development. Herein, we present the functional and molecular relevance of the canonical NF-κB p65 subunit in nasopharyngeal carcinoma (NPC). Loss- and gain-of-function approaches were utilized to reveal the functional characteristics of p65 in propagating tumor growth, tumor-associated angiogenesis, and epithelial-to-mesenchymal transition in NPC cells. Extracellular inflammatory stimuli are critical factors that trigger the NF-κB p65 signaling; hence, we investigated the components of the tumor microenvironment that might potentially influence the p65 signaling pathway. This led to the identification of an extracellular matrix (ECM) protein that was previously reported as a candidate tumor suppressor in NPC. Our studies on the Latent Transforming Growth Factor-β Binding Protein 2 (LTBP2) protein provides substantial evidence that it can modulate the p65 transcriptional activity. Re-expression of LTBP2 elicits tumor suppressive effects that parallel the inactivation of p65 in NPC cells. LTBP2 was able to reduce phosphorylation of p65 at Serine 536, inhibit nuclear localization of active phosphorylated p65, and impair the p65 DNA-binding ability. This results in a consequential down-regulation of p65-related gene expression. Therefore, the data suggest that the overall up-regulation of p65 expression and the loss of this candidate ECM tumor suppressor are milestone events contributing to NPC development.

## Introduction

The NF-κB protein family comprises of RelA (p65), RelB, c-Rel, p50 (p105 precursor), and p52 (p100 precursor) [1]. They are characterized by a Rel homology region that allows the subunits to bind DNA, interact with its inhibitory proteins (IκB proteins), and form homo- or hetero-dimeric complexes with each other [2,3]. Amidst intense research on the Rel family proteins, much of our understanding is derived from its evolutionarily conserved immunological signaling pathway. However, there is mounting evidence of its diverse biological roles in influencing gene expression, cell survival, differentiation, and proliferation [4], while its dysregulation will ultimately lead to disease causation, and notably, cancer [5–7].

The NF-κB pathway has been closely associated with nasopharyngeal carcinoma (NPC). In the past decade, studies by a number of investigators concur that NF-κB plays an important role in NPC development [8–11]. However, to date, limited reports have presented the functional and molecular aspects of the canonical p65 subunit of NF-κB in NPC. The functional role of this important cancer-associating protein is still largely unclear in NPC. Herein, we provide comprehensive evidence using genetic manipulation and functional assay approaches to show that the p65 subunit is indeed a critical player in NPC. In addition, our studies also identified a possible regulator of p65, which is a putative extracellular matrix (ECM) tumor suppressor gene (TSG), *LTBP2*, [12] mediating its anti-tumorigenic effects.

The ECM is a major component of the tumor microenvironment, which plays a highly dynamic and complex role in tumor development and progression. It modulates the interactions between extracellular and intracellular circuitry and coordinates cell communication networks within a tumor mass [13]. Deregulated crosstalk between tumor cells and the ECM may release cells from a dormant state, and thereby, lead to detrimental outcomes such as metastasis [14,15]. The ECM forms a vital structural scaffold within all tissues and organs, while providing the essential biochemical and biomechanical signals that stimulate and direct tissue morphogenesis, differentiation, and homeostasis [16]. Its intricacy and importance can only be appreciated by understanding the diseases that arise when the ECM fails to serve its physiological purpose [17,18].

Our previous studies identified an ECM protein, *LTBP2*, as candidate TSG in NPC, since it has demonstrated significant suppressive effects on NPC tumorigenesis. LTBP2, a 240 kDa extracellular glycoprotein mapping to chromosome 14q24, belongs to a family of latent TGF-β binding proteins (LTBPs) that regulates a well-known growth factor, TGF-β [19,20]. Unlike other family members, LTBP2 does not bind directly to latent TGF-β in the ECM, hence, highlighting its obscure functions. LTBP2 has been characterized as a regulator of cell adhesion and elastic fiber assembly by binding to DANCE/fibulin-5 [21,22]. In cancer, Vehvilainen *et al* [23] studied the adhesion function of LTBP2 in melanoma cells, while we showed the pleiotropic anti-tumorigenic characteristics of LTBP2 in esophageal squamous cell carcinoma (ESCC) and NPC through comprehensive functional assay approaches [12,24]. Prior study has also shown that *LTBP2* was down-regulated in all NPC cell lines, attributing to epigenetic modification of the gene promoter region [12]. To date, there are no reports on the downstream signaling cascade of LTBP2.

Nevertheless, the genetic basis of this understudied gene remains elusive. This study aims to 1) provide insights into the pathological implications and the underlying molecular mechanisms of the NF-κB p65 subunit in NPC; 2) investigate the biochemical pathways of *LTBP2* and examine its modulatory effects on the oncogenic activities of the NF-κB subunit. Therefore, our finding highlights the significance of the well-established canonical NF-κB pathway in NPC and demonstrates, for the first time, the communication network between an ECM protein and its impact on the intracellular signaling cascade in NPC.

## Materials and Methods

### Cell lines and culture

The NPC cell line, HONE1, was established from a biopsy specimen from a poorly differentiated squamous cell carcinoma of the nasopharynx [25,26]. HK1 was established from a recurrent well-differentiated squamous carcinoma of the nasopharynx of a Chinese male [27]. Both cell lines were cultured as described previously. Puromycin (InvivoGen, San Diego, CA, USA) was used as a selection drug for the virally transduced cells. Human umbilical vein endothelial cells (HUVEC, Cascade Biologics, Portland, OR, USA) were cultured as described [28]. Cell lines used in this study were genotype authenticated and screened for mycoplasma contamination.

### Lentiviral vectors and virus infection

The *LTBP2* and red fluorescent protein (RFP)-tagged *p65* wild type (WT) genes were cloned into the lentiviral vector backbone, pLVX-puro with an EF1a promoter. *IκBa-*super repressor (SR) gene was cloned into the pWPI lentiviral backbone. The p65- and IκBa-shRNA knockdown oligonucleotides, designed according to the RNAi consortium library [29], were cloned into pLKO.1 TRC cloning vector (Addgene plasmid 10878). The sequences of the two shRNA oligonucleotides are shown in S1 Table. pLKO.1-scramble shRNA vector (Addgene 1864) was used as a negative control. The *IκBa-*SR with three HA-tags was obtained from Addgene (24143) and subcloned into the pWPI system (Addgene plasmid 12254). The co-transfection system consisted of the respective gene construct, packaging vectors (pCMV-ΔR-8.74 or psPAX2), and the vesicular stomatitis virus G-protein envelope vector (pMD2G). The construct, packaging vector, and envelope vector were transfected into HEK 293T cells at a ratio of 4:3:1 using Fugene HD transfection reagent (Promega, Madison, WI, USA) according to the manufacturer’s instructions.

### Western blot analysis

Western blot analyses were performed as described previously [30]. Cultured NPC cell lines were harvested at 70–80% confluence and lysed using RIPA buffer supplemented with protease inhibitors. The list of antibodies used in this study is summarized in S2 Table.

### Subcellular fractionation

Cell lysates were prepared using non-denaturing cell lysis buffer supplemented with protease inhibitors. Cell organelles were then separated by ultracentrifugation to obtain nuclear and cytoplasmic/membrane fractions as described previously [31].

### 2-Dimensional (2D) and 3-Dimensional (3D) colony formation assay

In the 2D colony formation assay, 3000 transduced cells were seeded onto 6-well plates in triplicate and allowed to grow over a span of two weeks. Cells were then fixed with 10% formalin (Sigma-Aldrich, St. Louis, MO, USA) and stained with 1x diluted Giemsa reagent (Sigma-Aldrich, St. Louis, MO, USA) to identify cell colonies. The 3D matrigel colony formation assay was performed as previously described [32]. The 96-well flat bottom plates (NUNC, Thermo Scientific, Waltham, MA, USA) were first coated with matrigel (BD Biosciences, San Jose, CA, USA) before being seeded with 200 transduced cells. Colonies were formed over a span of two weeks.

### Wound healing

The wound healing assay was performed as described [32]. The percentages of wound closure were calculated based on the formula: (migrated distance of cells after 24 h ÷ initial distance of the wound at 0 h) x 100. All wound healing experiments were conducted in triplicates and the average percentage wound closure was taken.

### Real-time cell migration assays

Real-time cell migration monitoring was conducted using the xCELLigence system (Roche Applied Science, Penzberg, Upper Bavaria, Germany) following the manufacturer’s protocol. In brief, 8x10^4^ cells were seeded into each well in the upper chamber of the CIM plate, which was coated with collagen. The lower chamber containing 10% serum media attracts the cells across the chambers, which will then be recorded real-time using a RTCA DP analyzer (Roche Applied Science, Penzberg, Upper Bavaria, Germany).

### Cell migration chamber assays

The migration assay was performed as reported previously [33,34]. In brief, 2 x 10^5^ HONE1 cells in serum-free medium were seeded in the top chamber of a BD BioCoat Control 8.0μm PET Membrane 24-well cell culture insert (BD Biosciences, San Jose, CA, USA). The bottom chambers were filled with serum-enriched medium that acts as a chemoattractant. Cells were subsequently stained after 24 hours (migration) with 1% crystal violet. Cells that underwent migration were counted using an inverted light microscope (Nikon TMS, Ontario, Canada) and SPOT software 4.6 (Diagnostic Instruments, Sterling Heights, MI, USA).

### Human umbilical vein endothelial cell assay

The tube formation assay was conducted as described [28] using vector-alone (VA) and LTBP2 or FBLN2-conditioned media. HUVEC was first serum-starved for one hour prior to being seeded with 4 x 10^4^ cells into each well of a flat bottom 96-well plate coated with 50μl of matrigel. Conditioned media (100μl) from the vector-alone (VA) or gene transfectants were then applied onto the cells and incubated for 6–8 hours.

### Quantitative reverse transcription-polymerase chain reaction

Quantitative RT-PCR analysis was performed on a Step-One Plus PCR machine (Applied Biosystems, Life Technology, Carlsbad, CA, USA) as described previously [35]. The SYBR green PCR core reagent kit (Applied Biosystems, Life Technology, Carlsbad, CA, USA) was used with gene-specific primers and *GAPDH*-specific primers as a control. Primers used are summarized in S3 Table.

### Immunohistochemistry (IHC)

IHC staining for the CD34 marker (1:100; Santa Cruz Biotechnology, Dallas, TX, USA) on progenitor cells of blood vessels was performed using the standard streptavidin-biotin-peroxidase complex method as previously reported [30].

### Immunofluorescence staining

Transduced HONE1 and HK1 cell lines were fixed in formalin for 10 min and permeabilized with 0.5% Triton X-100 in PBS as described [33]. The phosphorylated p65 Serine 536 primary antibody (1:100) was incubated with the cells overnight at 4°C, followed by a FITC-conjugated goat anti-rabbit secondary antibody (Sigma-Aldrich, St. Louis, MO, USA) (1:100). Total IκBa was labeled with Alexa Fluor 546 goat anti-mouse secondary antibody (Molecular Probes, Life Technology, Carlsbad, CA, USA). Nuclei were stained with DAPI, cells were mounted onto glass slides with Slowfade Gold Antifade Reagent (Molecular Probes) and viewed under a fluorescence inverted microscope (Nikon Eclipse T*i*, Nikon Instruments, Melville, NY, USA) or confocal microscope (Carl Zeiss LSM 710, Zeiss Microscopy, Jena, Thuringia, Germany).

### NF-κB p65 chemiluminescence transcription factor binding assay

The NF-κB/p65 transcription factor binding assay was carried out using the Millipore Universal EZ-TFA Chemiluminescent Transcription Factor Kit following the manufacturer’s protocol (Millipore, Billerica, MA, USA).

### *In vivo* tumorigenicity assay

*In vivo* experiments were conducted as reported previously [36]. Cells were administered via subcutaneous injection into both flanks of the female BALB/cAnN-nu (nude) mice. *In vivo* tumor growth was monitored by measuring the tumor mass at weekly intervals. A total of 1 x 10^7^ cells/site was used for HONE1 and for HK1 cells. Matrigel plug tumors were removed after one week for IHC staining, while typical tumor mass was monitored over a 2–3 week period. The mice were sacrificed by cervical dislocation at the end of the experiment. *In vivo* studies were conducted with a valid license under the Animals (Control of experiments) Ordinance from the Department of Health (Hong Kong). The study was specifically approved by the Committee on the Use of Live Animals in Teaching and Research (CULATR) of the University of Hong Kong (Approval number: 2501–11).

### Statistical analysis

Student’s t-test was performed for all statistical analyses unless stated otherwise. *P*-values < 0.05 were considered significant and error bars represent the standard error mean.

## Results

### NF-κB p65 inactivation inhibits tumorigenicity *in vitro* and *in vivo*

The p65 activities were attenuated by utilizing two independent loss-of-function approaches, i.e. p65 shRNA knockdown and over-expression of IκBα-super repressor (SR) (S1A Fig) in NPC cells.

**Fig 1:**
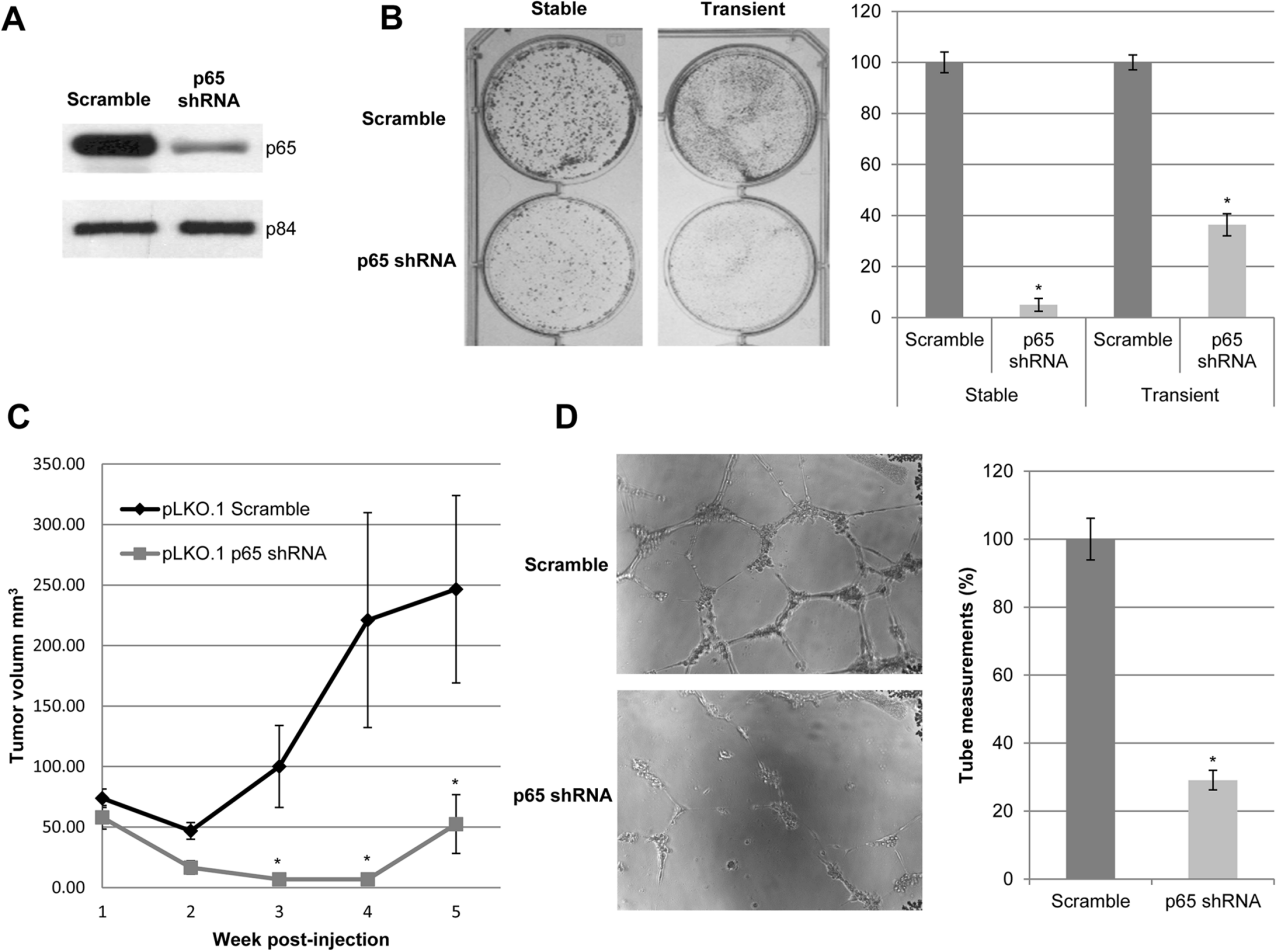
Knockdown of p65 reduces the abilities of *in vitro* colony formation, *in vivo* tumor formation, and angiogenesis in NPC. **(A)** Western blot analysis shows the transient shRNA-mediated knockdown of p65 in HONE1 cells using shRNA. The p84 serves as a loading control. **(B)** Two-dimensional (2D) colony formation assay shows that stable and transient knockdown of p65 reduces the number of colonies formed compared to scramble-transduced HONE1 cells. Data represented on the bar graph is the average of triplicate experiments ± S.E.M. **(C)** Nude mice were inoculated subcutaneously with p65 shRNA and scramble HONE1 cells. p65 shRNA-transduced cells showed delayed and reduced tumor growth compared to scramble control cells. Each data point represents an average tumor volume of six injection sites inoculated for each cell population ± S.E.M. **(D)** HUVEC tube formation is abrogated with p65 shRNA conditioned medium compared to the scramble control conditioned medium from HONE1 cells. The bar charts indicate data obtained from an average of triplicate experiments ± S.E.M. The (*) indicates P-value < 0.05 for all graphs.

Successful p65 knockdown by p65 shRNA was first verified (Fig 1A and S1B Fig). Diminished colony numbers in *in vitro* colony formation assay (Fig 1B) and reduced *in vivo* tumor growth kinetics in nude mouse models (Fig 1C) proved that the knockdown of p65 elicits tumor suppressive effects. In addition, the gene expression level of NF-κB related genes such as *MMP3*, *SOX9*, *ICAM*, *MCAM*, *EGFR*, and *FN1*, were down-regulated upon knocking down p65 (S1C Fig)

**Fig 2:**
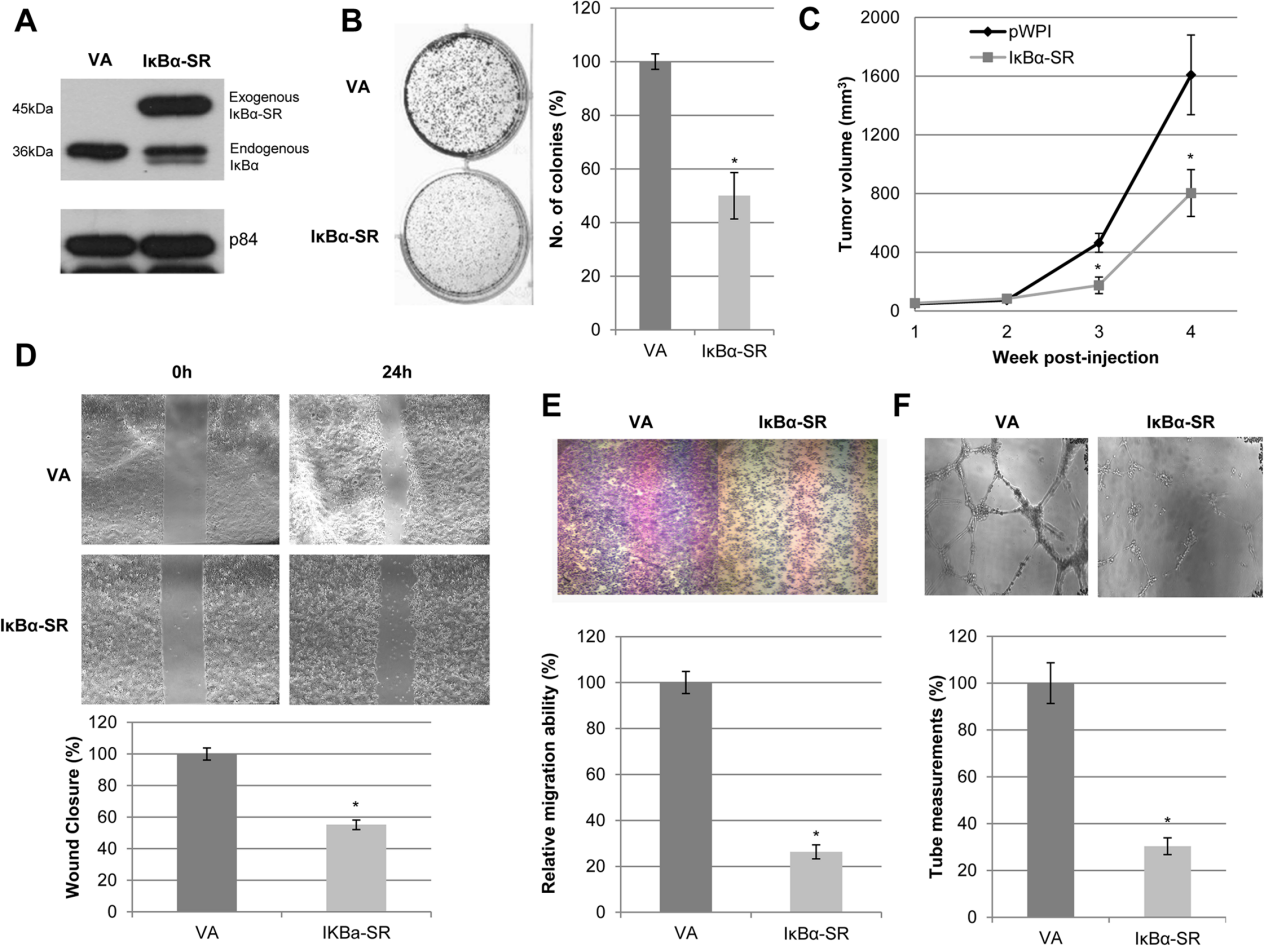
Inactivation of p65 reduces the abilities of *in vitro* colony formation, *in vivo* tumor formation, migration, invasion, and angiogenesis in NPC. **(A)** Western blot analysis shows the expression of exogenous IκBα-SR with triple HA-tag (^) in HONE1 cells. The p84 served as a loading control. **(B)** 2D colony formation assay shows that IκBα-SR suppressed the colony-forming ability of HONE1 cells compared to pWPI-vector alone (VA). Bar graphs indicate data obtained from an average of triplicate experiments ± S.E.M. **(C)** Nude mice were inoculated subcutaneously with IκBα-SR and pWPI-VA HONE1 cells. IκBα-SR-transduced cells showed delayed and reduced tumor growth kinetics compared to pWPI-VA. Each data point represents an average tumor volume of six injection sites inoculated for each cell population ± S.E.M. **(D)** Wound healing analysis for pWPI-VA and IκBα-SR showed delayed migration of IκBα-SR cells compared to VA. Bar graphs show the percentage difference between IκBα-SR and VA cells ± S.E.M. **(E)** Migration chamber assays showed that IκBα-SR-expressing HONE1 cells reduced migration compared to VA cells. Data represented on the bar graph are the average of triplicate experiments ± S.E.M. **(F)** HUVEC tube formation was suppressed with IκBα-SR conditioned medium compared to that of VA cells. Data represented on the bar graph are the average of triplicate experiments ± S.E.M. The (*) for all graphs indicate P-value < 0.05.

On the other hand, IκBα-SR was over-expressed in NPC cells, as illustrated by Western blots (Fig 2A). The exogenous IκBα-SR preferentially resides in the cytosol (S1D Fig), which is analogous to the normal biological localization of endogenous IκBα. Expression of the IκBα-SR resulted in significant reduction of colony size and numbers (Fig 2B). *In vivo* mouse models further confirmed the suppressive effects of IκBα-SR (Fig 2C). Furthermore, IκBα-SR-expressing cells exhibited a slower migratory potential compared to the pWPI-VA (Fig 2D and 2E), suggesting that p65 plays an important role in regulating cell motility in NPC. Both indirect and direct inactivation of p65 rendered consistent experimental results, thus, providing solid evidence that p65 is potentially a good target for impeding the growth of NPC cells.

### The p65 pathway is involved in tumor-associated angiogenesis by regulating the pro-angiogenic factors in NPC

The HUVEC tube-formation assay was utilized to examine the pro-angiogenic activity of p65 in NPC. Conditioned media were collected from cells of p65 shRNA knockdown, over-expression of IκBα-SR, and their corresponding controls to perform HUVEC tube formation assay. HUVEC tube-forming abilities were largely suppressed by conditioned media collected from p65 shRNA knockdown- and IκBα-SR- expression (Figs 1D and 2F), possibly due to a reduction in the quantities of pro-angiogenic factors in the media. To further confirm that the anti-angiogenic activities were attributed to the inactivation of p65, expression levels of pro-angiogenic factors were examined (S1B Fig). The down-regulation of p65 (*RELA*) expression in p65 knockdown cells was confirmed, when compared with the scramble control cells. IκBα (*NFKBIA*), a well-established p65-regulated negative feedback protein, was used as a direct indicator of p65 activity. Since *NFKBIA* expression was diminished after p65 knockdown, we then screened a panel of NF-κB angiogenic-related target genes that include *IL6*, *IL8*, *RANTES*, *MCP1*, *VEGF165*, *VEGF189*, and Total *VEGF*. Taken together, these findings revealed that the inactivation of p65 in NPC tumor cells reduced the secretion of pro-angiogenic factors, resulting in inhibition of angiogenesis.

### Enhanced p65 signaling triggers *in vitro* tumorigenic responses

A two-prong approach in analyzing p65 gain-of-function abilities was conducted. The effects of p65 activation in NPC were probed by RFP-fusion p65 over-expression and IκBα knockdown, to eliminate the p65 negative feedback mechanism.

**Fig 3:**
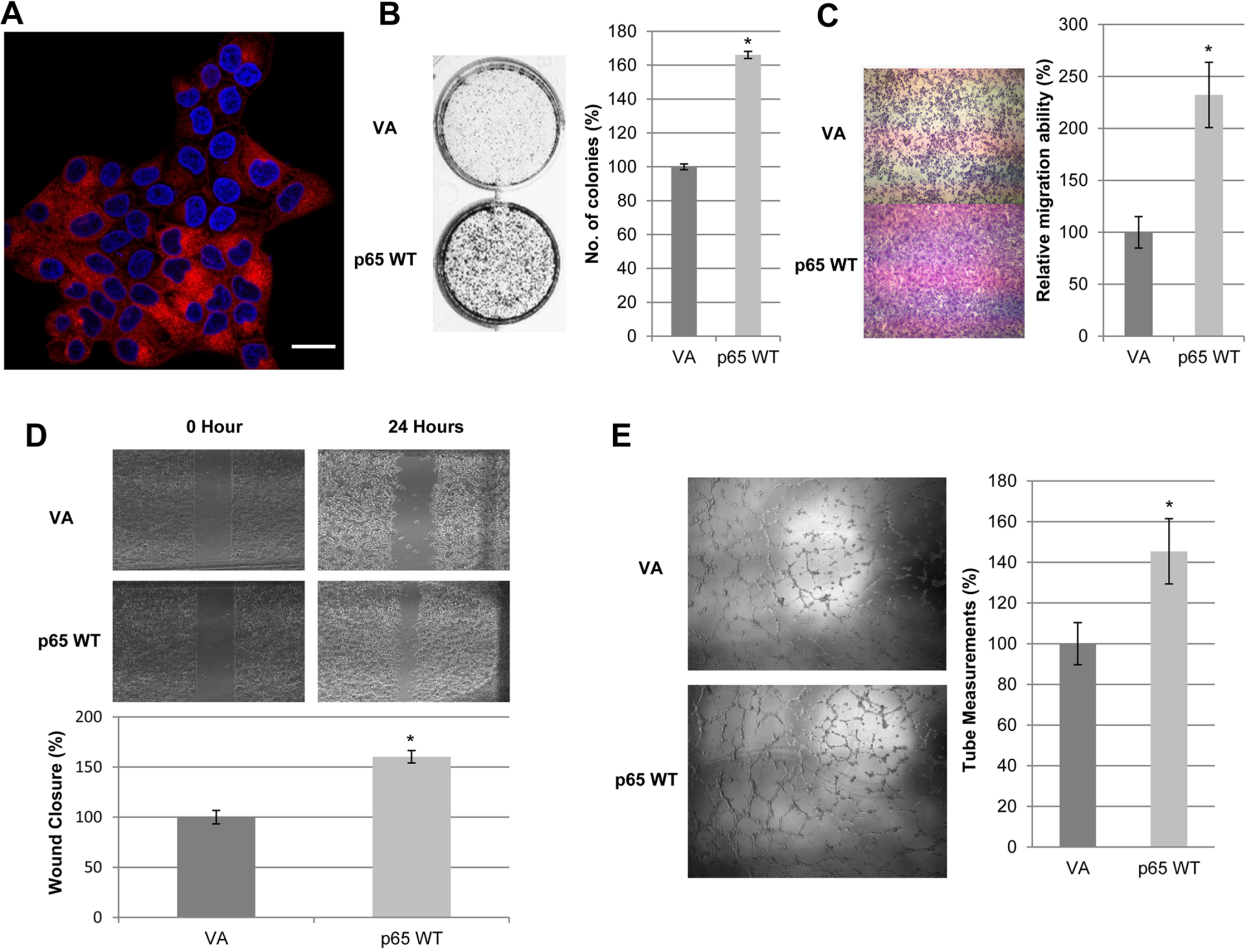
Enhanced p65 expression promotes *in vitro* tumorigenic responses. **(A)** Confocal visualization of cytolocalization of RFP-fusion p65 WT (63x magnification). Scale bar represents 1μm. **(B)** 2D colony formation assay showed increased number of colonies in p65 WT-overexpressing cells compared to RFP-tagged pLVX-VA cells. Bar graphs show the percentage difference between the number of colonies in p65 WT and VA cells ± S.E.M. **(C)** Migration chamber assays showed that p65 WT-overexpressing HONE1 cells enhanced migration compared to VA cells. Bar graphs illustrate the percentage difference between the relative migration abilities of p65 WT and VA cells ± S.E.M. **(D)** Wound healing analysis for pLVX-VA and p65 WT showed an increase in migratory potential of p65 WT transduced cells compared to VA. Bar graphs show the percentage difference between p65 WT and VA cells ± S.E.M. **(E)** HUVEC tube formation was augmented with p65 WT conditioned media compared to that of VA cells. Bar graphs illustrate the percentage difference between the tube measurements of p65 WT and VA conditioned media-induced HUVEC cells ± S.E.M. The above data were obtained from three independent experiments and the (*) for all graphs indicate P-value < 0.05.

The exogenous RFP-p65 was mainly detected in the cytoplasmic region (Fig 3A). The p65 subunit indeed does appear to promote tumorigenicity, since functional assays showed that HONE1 RFP-p65 WT-expressing cells possess superior colony-forming abilities (Fig 3B), increased migration (Fig 3C and 3D), and enhanced angiogenesis (Fig 3E).

It is widely known that the endogenous IκBα acts as a tightly-controlled and highly sensitive feedback mechanism to regulate p65 activities [37]. Hence, to further understand the function of p65 in NPC, we sought to knockdown IκBα, so as to eliminate its feedback response. Angiogenic and EMT induction abilities were examined after IκBα inactivation by determining the expression of a panel of angiogenic factors that include *IL6*, *IL8*, Total *VEGF*, *VEGF165*, *VEGF189*, *PDGFB*, and *MCP1*. IκBα knockdown caused a dramatic up-regulation of *IL6* and *IL8* expression of up to approximately 41-fold. Expression of Total *VEGF*, *VEGF165*, *VEGF189*, and *MCP1* was elevated by more than 2-fold, whereas *PDGFB* only showed a slight increase (S2 Fig). In addition, epithelial markers *CDH1*, *CTNNA1*, and *CTNNB1* were down-regulated by approximately 2-fold, whilst mesenchymal markers *SNAI2*, *SNAI1*, *TWIST1*, *VIM*, *smooth muscle-actin (ACTA2)*, and *SOX9* were significantly up-regulated, and *CDH2* expression was slightly increased. (S3A Fig). Correspondingly, the cell morphology of HONE1 was altered as these epithelial cells undergo mesenchymal transition (S3B Fig).

### p65-mediated molecular signaling regulates expression of EGFR and EMT markers

**Fig 4:**
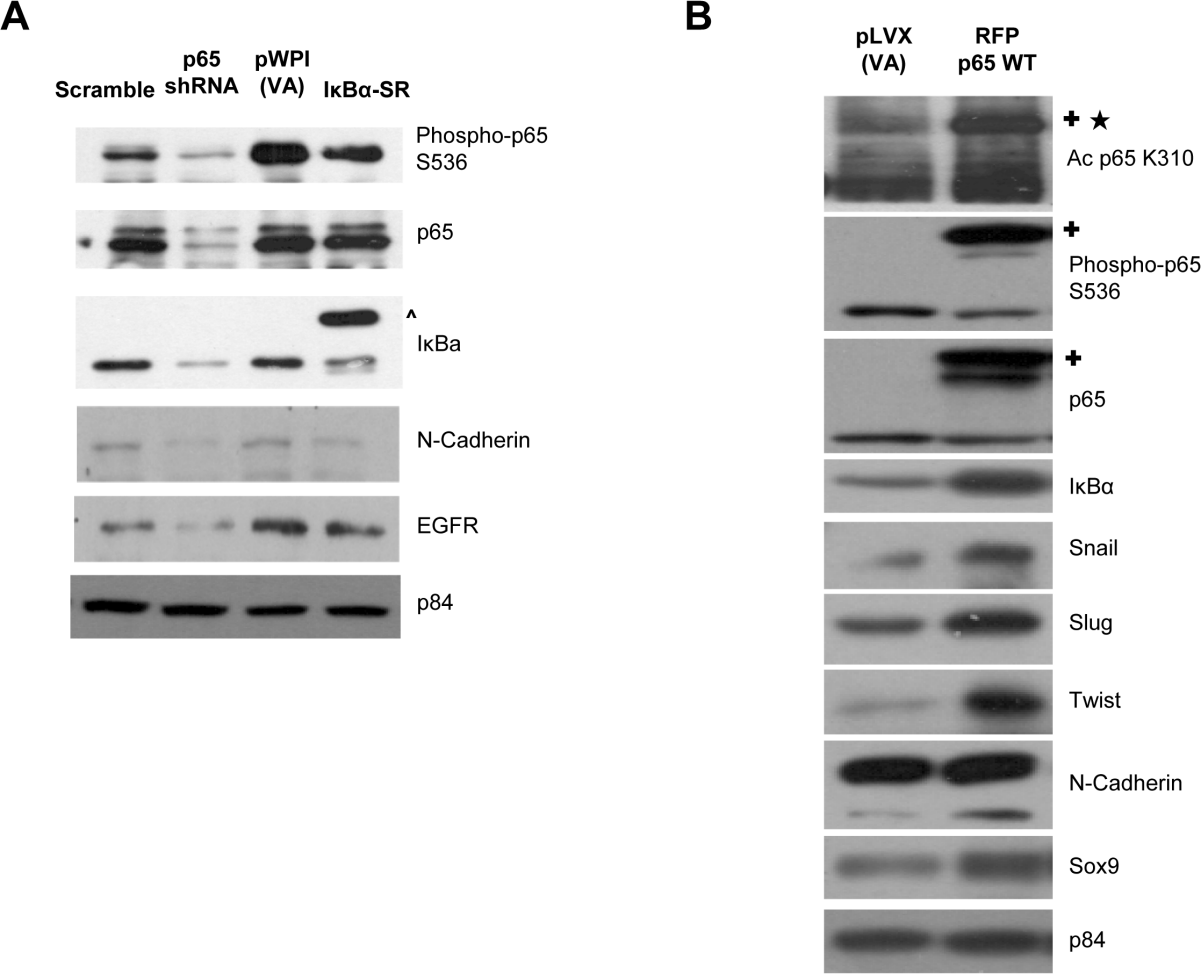
p65 signaling pathway regulates the protein expression of EGFR and EMT markers. **(A)** Western blot analysis of p65shRNA- HONE1 cells showed reduced levels of phosphorylated p65 S536, total p65, and total IκBα compared to scramble control. IκBα-SR- HONE1 cells only showed a decreased amount of phosphorylated p65 S536, but did not affect the overall amount of total p65 when compared to pWPI-VA. Endogenous and exogenous IκBα are labeled as shown. The p84 was used as a loading control for scramble and p65 shRNA, pWPI-VA and IκBα-SR, separately. Protein expression of N-cadherin and total EGFR were all reduced in p65 shRNA and IκBα-SR HONE1 cells compared to scramble and pWPI-VA control. The (**^**) indicates the exogenous 3xHA-IκBα-SR. **(B)** Western blot analysis of p65 WT-overexpressing HONE1 cells showed increased acetylation at K310 and enhanced phosphorylation at S536 compared to VA cells. The p65 WT overexpression increases the protein levels of IκBα, snail, slug, twist, N-cadherin, and sox9, compared to VA cells. The p84 was used as a loading control. The (✚) indicates the exogenous RFP-p65 WT. The (★) indicates the acetylation band in the exogenously expressed RFP-p65 WT.

Besides functional approaches, the p65-mediated molecular signaling pathway alterations in NPC were assessed. Fig 4A shows that the over-expression of IκBα-SR only inhibited the phosphorylation of p65 at S536, but did not have significant effect on total p65 expression. This indicates that the IκBα-SR was able to attenuate p65 transcriptional activity without compromising its overall expression. Nonetheless, both p65 knockdown and IκBα-SR reduced the expression of N-Cadherin and EGFR (Fig 4A). Conversely, over-expression of RFP-fusion p65 WT induced significant IκBα up-regulation, hence, suggesting that the exogenous p65 proteins are transcriptionally active in HONE1 (Fig 4B). Moreover, exogenous p65 WT protein showed detectable acetylation at the lysine K310 site, while phosphorylation of endogenous p65 at S536 was reduced (Fig 4B). This could possibly be due to the increased expression of IκBα, which serves to regulate the amount of active phosphorylated-p65 in the cell. In addition, both long and short isoforms of N-Cadherin, and other EMT-related protein expression such as Snail, Slug, Twist, and Sox9 were elevated following the expression of exogenous p65. Taken together, these results further support the positive and negative regulatory impact of p65 on EMT-related protein expression (Fig 4B).

### LTBP2 disrupts the activation, nuclear translocation and subsequent transcriptional activity of NF-κB p65 subunit

The functional and molecular studies suffice to show that the oncogenic p65 requires important upstream regulatory mechanisms to modulate its detrimental outcomes in NPC development. The candidate ECM tumor suppressor protein, LTBP2, has emerged as a key p65 regulator that has been reportedly lost in all NPC cell lines [12].

**Fig 5:**
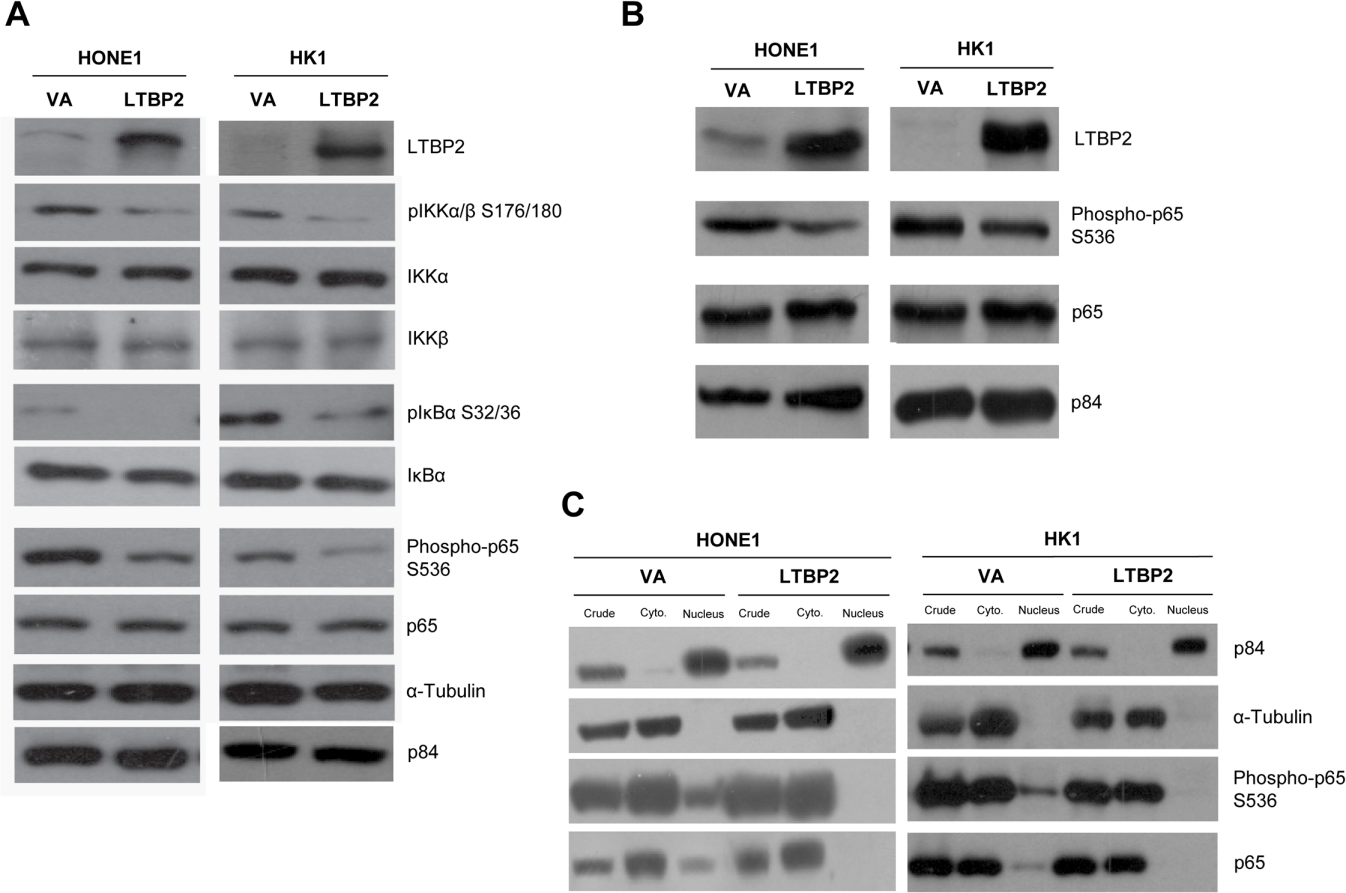
LTBP2 regulates the p65 signaling pathway. **(A)** Phosphorylated IKKα/β S176/180 and phosphorylated IκBα SS32/36 are also reduced in LTBP2-infected cells. Phosphorylated p65 serine 536 level is reduced in LTBP2-infected cells compared to VA in HONE1 and HK1. The p84 and α-tubulin were used as loading controls. **(B)** Matrigel plug tumors show a reduction in phosphorylated p65 S536 in LTBP2-transduced cells compared to VA. The p84 was used as loading control. **(C)** Subcellular fractionation results show that phosphorylated p65 S536 was reduced in the nuclear fraction of LTBP2-transduced cells compared to VA. The p84 was used as control to determine nuclear fraction, while α-tubulin was used as the control for the cytoplasmic fraction.

Phosphorylation of p65 was found to be reduced in *LTBP2*-re-expressing NPC cell lines (Fig 5A). Phosphorylated IKKα/β, which is known to directly phosphorylate p65 at one of the key serine 536 activation sites [38–40] and the inhibitors of NF-κB (IκBα) [41], were also reduced (Fig 5A). A reduction of phospho-IκBα may result in less IκBα degradation, hence allowing the inhibitor to couple to p65 and suppress its transcriptional activities (Fig 5A). Moreover, a reduced phospho-p65 level was also observed in Matrigel plugs containing LTBP2-expressing HONE1 and HK1 cells (Fig 5B). In particular, total p65 and phospho-p65 were reduced in the nuclear fraction, as determined after subcellular fractionation analysis (Fig 5C).

**Fig 6:**
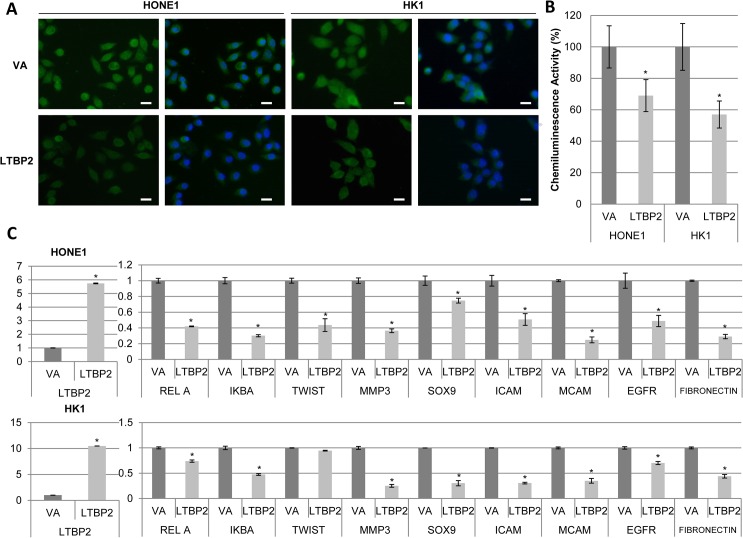
LTBP2 regulates the translocation and activities of p65. **(A)** Immunofluorescence staining of phosphorylated p65 S536 (green fluorescence) revealed weaker staining intensity as well as decreased nuclear staining in LTBP2-infected cells compared to VA. Nuclei were stained with 4, 5-diamidino-2-phenylindole (DAPI, blue fluorescence). Scale bar represents 5μm. **(B)** NF-κB binding reporter assay showed reduced chemiluminescence intensity in the nuclear fraction of LTBP2-transduced cells compared to VA in both HONE1 and HK1. Data represented on the bar graph are the average of triplicate experiments ± S.E.M. **(C)** Real-time PCR of NF-κB target genes showed that the transcriptional activities of *LTBP2*-infected cells were reduced compared to VA in both HONE1 (top) and HK1 (bottom). The panel of genes include: *RELA*, *I*κ*B*α, *TWIST*, *MMP3*, *SOX9*, *ICAM*, *MCAM*, *EGFR*, and *FN1*. *GAPDH* was used as an internal control. The above data were obtained from three independent experiments done in duplicate and the (*) for all graphs indicate P-value < 0.05.

Immunofluorescence staining of the phospho-p65 showed that the nuclear staining of phospho-p65 was also visibly weaker in *LTBP2*-re-expressing cells compared to VA (Fig 6A). Utilizing a chemiluminescence reporter assay specific for NF-κB p65 activity, the DNA binding abilities of p65 are substantially reduced in *LTBP2*-expressing HONE1 and HK1 cells (Fig 6B). These data are consistent with our earlier signaling pathway analysis (Fig 5A). To confirm the loss of p65 transcriptional activity, expression levels of various NF-κB target genes were interrogated. The panel of genes studied includes *RelA* (p65), *NFKBIA* (IκBα), *TWIST1*, *MMP3*, *SOX9*, *ICAM*, *MCAM*, *EGFR*, and *FN1*. All target genes were shown to be significantly down-regulated after re-expression of LTBP2 (Fig 6C). This suggests the importance of this candidate tumor suppressor gene in the regulation of the NF-κB signaling.

### LTBP2 suppresses cancer cell growth *in vitro* and *in vivo*

**Fig 7:**
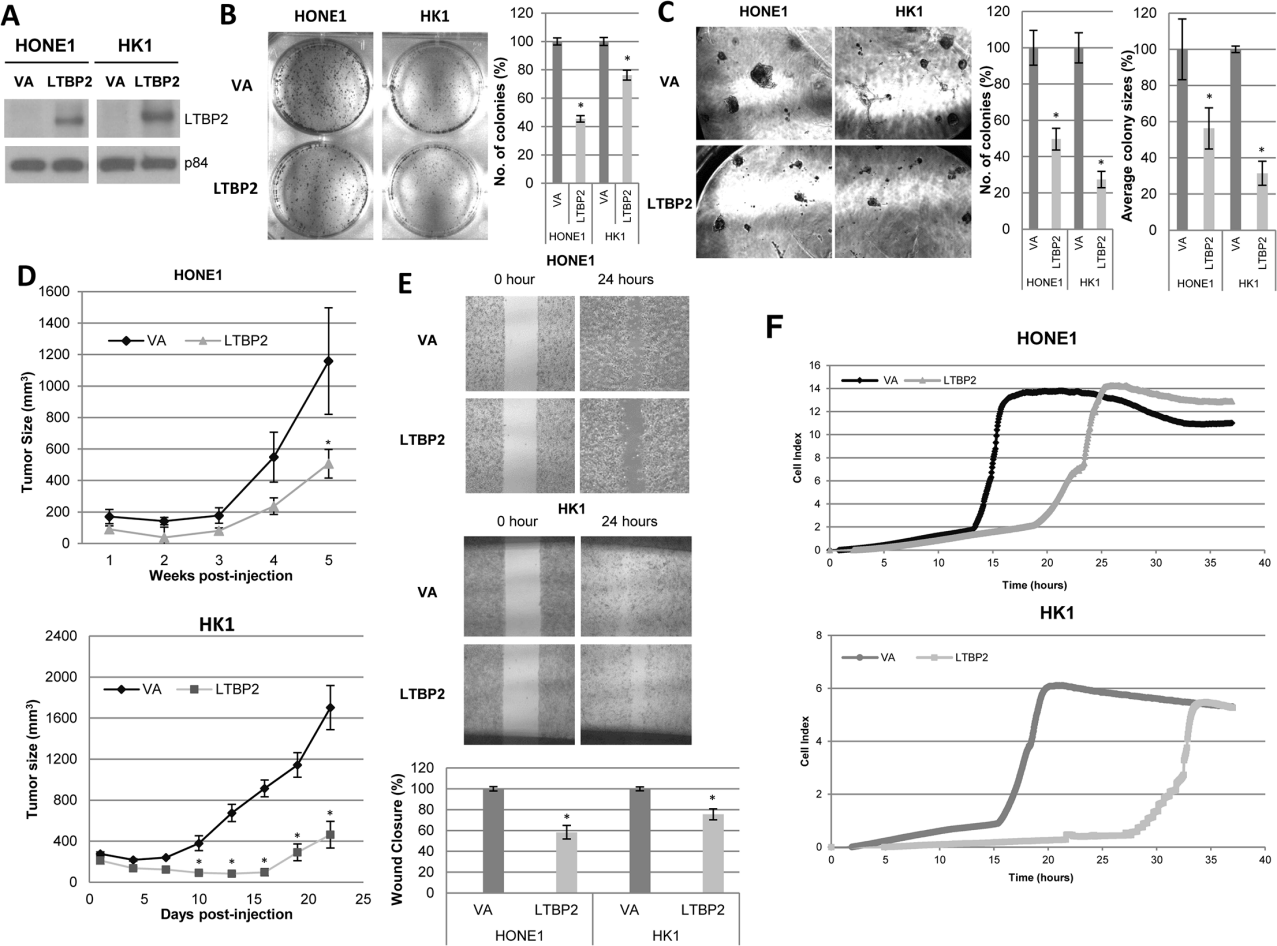
LTBP2 suppresses tumorigenic properties *in vitro* and *in vivo*. **(A)** Restoration of LTBP2 expression in HONE1 and HK1 cells using the lentiviral system, as detected by Western blot analysis. The p84 was used as a loading control. **(B)** 2D colony formation assay showed reduced number of colonies in *LTBP2*-expressing cells compared to pLVX-VA cells. Bar graphs show the percentage difference between number of colonies in LTBP2 and VA cells ± S.E.M. **(C)** 3D colony formation assay showed reduced number and size of LTBP2 cell colonies compared to VA. Percentage difference is represented by the bar graphs ± S.E.M. **(D)** *In vivo* tumor growth kinetics of LTBP2 and VA HONE1 and HK1 cells. Each data point represents an average tumor volume of six injection sites inoculated for each cell population ± S.E.M. **(E)** Wound healing analysis for HONE1 and HK1 cell lines showed delayed migration of LTBP2-transduced cells compared to VA. Bar graphs show the percentage difference between LTBP2 and VA cells ± S.E.M. **(F)** Real-time monitoring of cell migration using xCELLigence RTCA DP analyzer in LTBP2-transduced HONE1 and HK1 cells. The above data were obtained from three independent experiments and the (*) for all graphs indicate P-value < 0.05.

LTBP2 was examined to determine its functional role in NPC cells. In order to study the function of this protein of interest, a lentivirus expression system was established to re-express *LTBP2* in LTBP2-silenced NPC cells, HONE1 and HK1 (Fig 7A). Re-expression of LTBP2 in NPC cell lines caused a reduction in the numbers and sizes of colonies in both 2D and 3D culture systems (Fig 7B and 7C). At an *in vivo* level, LTBP2 greatly suppressed the tumorigenicity of HONE1 and HK1 NPC cell lines, giving rise to significantly smaller tumors compared to the control group (Fig 7D). This further confirms the role of the NF-κB inactivation to suppress NPC tumorigenesis.

### LTBP2 exhibits regulatory roles in migration and angiogenesis

To assess whether LTBP2 also inhibited other hallmarks of tumorigenesis, the function of LTBP2 in migration and angiogenesis was probed.

Re-expression of LTBP2 in NPC cells impeded its migratory potential as measured by the wound healing assay (Fig 7E). To further substantiate our findings, an xCELLigence RTCA DP analyzer was then utilized to quantify the degree of migration delay after re-expression of LTBP2 in NPC cells. Results suggested that the re-expression of LTBP2 hampered the rate of migration, when compared to VA-infected cells (Fig 7F).

**Fig 8:**
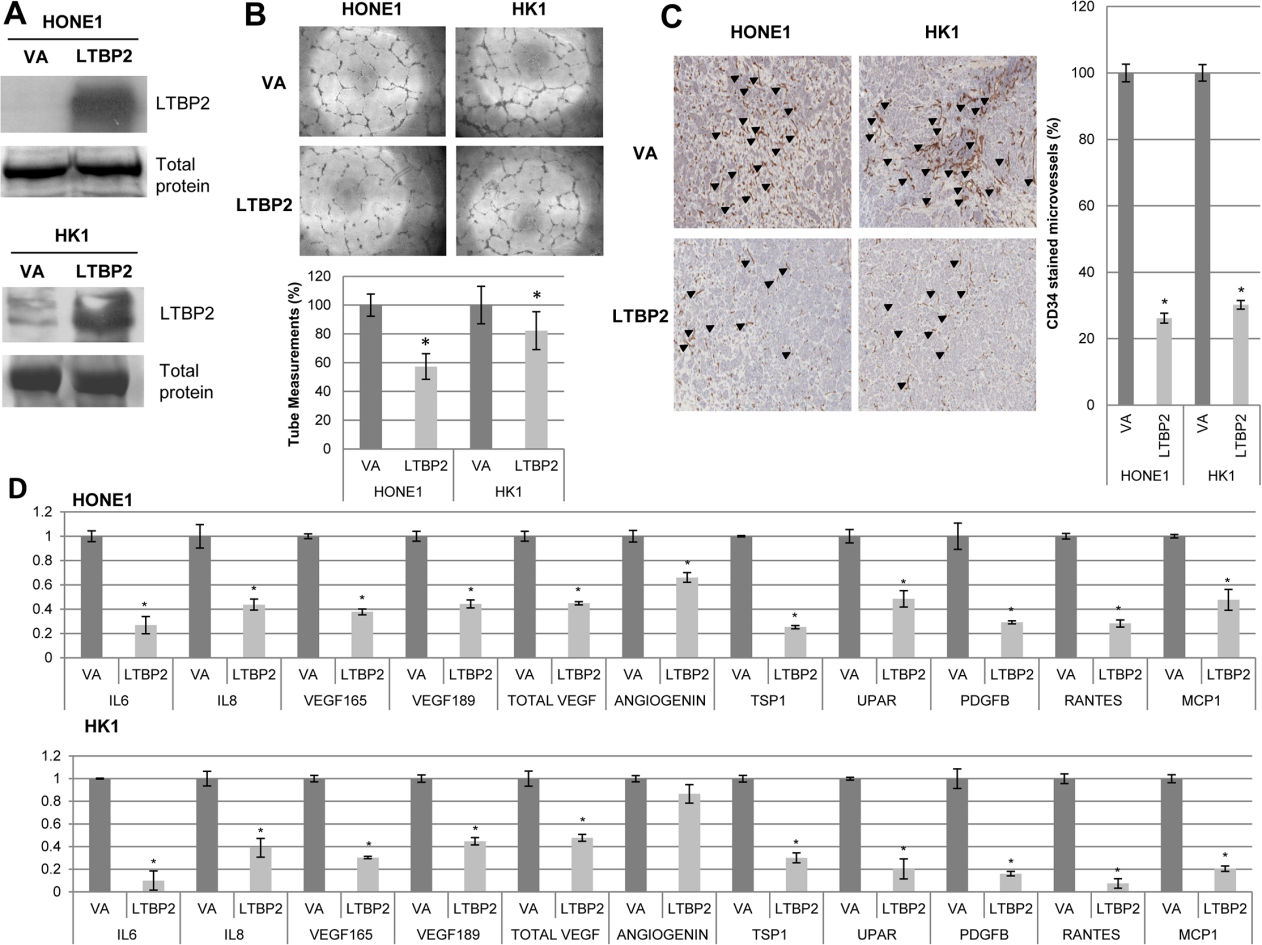
LTBP2 reduces angiogenesis *in vitro* and *in vivo*. **(A)** Expression of secreted LTBP2 in conditioned media from HONE1 and HK1 LTBP2-transduced cells. Coomassie blue staining of total protein in conditioned media was used to indicate equal loading. **(B)** LTBP2-conditioned media from HONE1 and HK1 cells suppressed the tube formation ability of HUVEC cultures. Bar graph represents an average of triplicate experiments ± S.E.M. **(C)** Matrigel plug tumors were stained for CD34 to visualize the microvessels, as indicated by the black arrows. LTBP2-transduced cells displayed significantly fewer microvessels in matrigel plugs compared to VA cells, as indicated by the bar graphs. **(D)** Real-time PCR of HONE1 (top) and HK1 (bottom) LTBP2- and VA-transduced cells showed the down-regulation of angiogenesis-related genes with LTBP2 expression: *IL6*, *IL8*, *VEGF 165*, *VEGF 189*, *total VEGF*, *ANG*, *TSP1*, *uPAR*, *PDGB*, *RANTES*, and *MCP1*. The housekeeping gene, *GAPDH*, was used as an internal control. The above data were obtained from three independent experiments in duplicate and the (*) for all graphs indicate P-value < 0.05.

The correlation of angiogenesis and the canonical NF-κB pathway has been well-documented in various cancers [2,42–48], including NPC [10,11,49,50]. The anti-angiogenic potential of LTBP2 was investigated. Conditioned media from the LTBP2-re-expressing NPC HONE1 and HK1 cells (Fig 8A) decreased *in vitro* HUVEC tube-forming ability (Fig 8B). The effect of LTBP2 in regulating angiogenesis *in vivo* was then determined by the standard nude mouse matrigel plug assay. After staining the matrigel plugs with a blood vessel marker, CD34, *LTBP2* re-expression induced a distinct reduction in the relative numbers of microvessels (Fig 8C). This can be explained by the down-regulation of various pro-angiogenic factors, *IL6*, *IL8*, *VEGF165* and *189*, Total *VEGF*, *ANG*, *TSP1*, *uPAR*, *PDGFB*, *RANTES*, and *MCP1* in LTBP2-expressing cells (Fig 8D). Collectively, these data confirm the importance of LTBP2 in suppressing tumor-associated angiogenesis in NPC through significant reduction of pro-angiogenic factors.

The corollary of these findings indicates that the ECM LTBP2 protein plays crucial roles in the tumor microenvironment and are implicated in the critical NF-κB p65 signaling cascade in NPC.

## Discussion

NPC, one of the most common malignancies in the Southern China region, is hampered in its diagnosis due to early age onset, obscure predisposition, and asymptomatic manifestations [51,52]. Nonetheless, the EBV infection in NPC was singled out as a major etiological factor with its undisputedly strong association between NPC and EBV [53–57]. This also highlights the immunological implication of such virally-induced epithelial cancers, as observed by many independent research teams over the years. Besides viral infection, other inflammatory stimuli such as prolonged exposure to carcinogens and/or mutagens will also drive pathologic changes in the tumor microenvironment that eventually promote tumor progression [8,9,58]. Chronic inflammation leads to a prolonged surge in pro-inflammatory cytokine release, as well as fibroblast and leukocyte activation through the innate and adaptive immune system. This will then stimulate the tumor microenvironment to undergo a complex cascade of molecular and biological changes in the ECM. NF-κB is a key player in mediating inflammatory responses and regulating key cytokines such as TNF-α, leading to cancer development [47,59–62]. Nevertheless, the direct role of the canonical NF-κB p65 subunit in NPC has remained largely elusive. Thus, we now provide *in vitro* and *in vivo* evidence of the importance of the p65 subunit in NPC.

Using the loss- and gain-of-function approaches, p65 was shown to play an important role in regulating cell growth and angiogenesis. Since direct shRNA-mediated knockdown may result in possible off-target effects, potentially causing false-positive phenotypic changes to cells [63,64], IκBα-SR and RFP-p65 WT were used to minimize off-target effects. IκBα-SR contains two mutations at serines 32 and 36, where serines were replaced by alanines as S32A/S36A [65] (S3A Fig). Thus, ubiquitination cannot occur at those two sites, resulting in a non-degradable IκBα that can sequester p65 in the cytoplasm and inhibit its transcriptional activity [37,66].

### p65 is a key driver of tumorigenicity in NPC

Aberrant manipulation of p65 expression has demonstrated that this transcription factor has direct effects on tumor growth and angiogenesis. Moreover, p65 activation clearly induces EMT, through the up-regulation of important EMT-related proteins such as Snail, Slug, Twist, N-Cadherin, and Sox9 with tumor cells, also increasing aggressiveness and acquiring the ability to metastasize. However, the p65 WT-overexpressing cells did not assume a fibroblast-like morphology (data not shown). This may be due to a robust negative feedback mechanism rendered by a marked increase in IκBα expression, and thus, hampering a complete morphological change [37]. This postulation was further corroborated by silencing the IκBα, that later resulted in complete EMT with the up-regulation of mesenchymal markers, down-regulation of epithelial markers, and the induction of cell morphological changes. A change in morphology is a typical characteristic of the EMT phenonmenon due to the loss of adheren junctions in the cells, causing cells to elongate and then metastasize [67].

### p65 is tightly regulated by the IκBα negative feedback mechanism and important ECM proteins in NPC

While it seems imperative for p65 to mediate its function in the nucleus, the latest controversy arises in NPC, due to the cytolocalization of p65. Ding *et al*. [68] showed detectable levels of nuclear phosphorylated p65 S536 with and without LMP1 and IκBα-SR induction. However, the lack of nuclear p65 IHC staining in NPC was reportedly correlated with poor prognosis of NPC [11]. Incidentally, they showed that cytoplasmic p65 was found in 100% of NPC clinical samples. Moreover, by knocking down the apparent cytoplasmic p65, the team also saw a suppression of cell migration and invasion abilities through reduced MMP9 expression and activity [49]. In contrast, the non-canonical NF-κB pathway is known to be constitutively active in NPC. RelB, Bcl3, and p50 were highly up-regulated, with both Bcl3 and p50 directly influencing EGFR expression [69–71].

Nevertheless, the direct roles of p65 in NPC have yet to be fully characterized. This current study provides evidence for the acetylation of the lysine K310 site on p65, as an indication of a fully trans-activated p65, when p65 WT is overexpressed. Detecting the acetylation on K310 is technically challenging, due to low basal level of this acetylation in our cell line system. Despite eliciting several cellular events with p65 WT over-expression, nuclear translocalization of p65 is rather infrequent and barely detectable. Our results are, therefore, in line with the findings that overexpressed p65 WT remains largely cytoplasmic [72]. Whether the nuclear translocation of p65 is necessary for oncogenic function, we deduce that a minor fraction of fully activated acetylated-K310 p65 WT can still enter the nucleus, and induce subsequent downstream gene expression of N-Cadherin, Twist, Slug, Snail, and Sox9. Consequently, we theorize that a trace amount of nuclear p65 is sufficient to promote tumorigenicity; p65 may have cytoplasmic functions that favor the cytoplasmic retention of p65 in NPC; cytoplasmic p65 expression is correlated with NPC progression; *de novo* expression of IκBα is an instant feedback mechanism that efficiently inactivates p65 in a temporal and spatial manner. These hypotheses are currently being tested in the lab.

In addition, the ECM proteins also play an important role in p65 regulation. Loss of vital ECM tumor suppressor genes alters the tumor microenvironment and promotes tumor growth and tumor-associated angiogenesis by modulating the intrinsic nuclear trafficking of the p65 subunit. Previously, we identified an ECM protein, LTBP2, as a candidate tumor suppressor in NPC. In-depth findings later confirmed that LTBP2 modulated the nuclear translocation of p65 and was particularly involved in anti-angiogenesis via the p65 signaling cascade. Secretion of pro-angiogenic cytokines by the tumor cells themselves remains germane for the sustenance of tumor-associated angiogenesis [73]. Hence, re-expression of LTBP2 ultimately led to a decreased expression of pro-angiogenic cytokines by NPC cells. Correspondingly, the anti-angiogenic effects induced by p65 inactivation approaches are akin to the re-expression of LTBP2. The down-regulation of these p65-mediated angiogenic factors: *VEGF*, *MCP1*, *PDGFB*, *RANTES*, *IL6*, and *IL8*, have all been widely reported [74–78].

More importantly, the analysis of *LTBP2* in tumor biopsies may hold potential prognostic or diagnostic value in a clinical perspective. We can postulate that the loss of LTBP2 may be one of the genetic hits in carcinogenesis based on the Knudson’s two-hits hypothesis [79]. Nevertheless, ongoing research is being conducted to validate this hypothesis

Finally, since NF-κB is primarily involved in the immunological signaling cascade mechanism originally found in B cells and T cells, the conundrum between these ECM proteins and their implication on tumor immunology and the microenvironment was investigated. Transcriptional activation of NF-κB requires various upstream signaling stimulants. In this study, we determined the potential role of *LTBP2* in regulating the NF-κB signaling cascade, particularly, the canonical p65 pathway. Our results showed that both proteins down-regulate the canonical p65 pathway through an as yet unknown mechanism. Additionally, LTBP2 exhibiting their anti-tumorigenic functions through the reduction of the active p65 transcription factor, was similar to the functional characteristics of p65 in NPC. In conclusion, the NF-κB p65 signaling pathway plays an important role in NPC development and provides potential therapeutic targets for NPC.

## Supporting Information

S1 Fig Inactivation of p65 in NPC cell lines. **(A)** Domain structure of IκBα-SR and IκBα are illustrated using the SMART 7 domain structure prediction tool. The mutations at serines 32 and 36 are shown in red **(B)** Real-time qPCR analysis of p65-related angiogenic genes include: *RELA*, *NFKBIA*, *IL6*, *IL8*, *RANTES*, *MCP1*, *VEGF165*, *VEGF189*, and Total *VEGF*. *GAPDH* was used as an internal control. p65 shRNA cells displayed reduced angiogenic gene expression compared with scramble control cells. **(C)** Quantitative PCR analysis of other p65-related NF-κB genes include: *MMP3*, *SOX9*, *ICAM*, *MCAM*, *EGFR*, and *FN1*. *GAPDH* was used as an internal control. p65 shRNA cells showed decreased expression of these genes compared to the scramble control cells. **(D)** Confocal microscopy of IκBα-SR transduced HONE1 cells showed the cytolocalization of exogenous and endogenous IκBα, mainly in the cytoplasmic region of the cells (red fluorescence staining). Scale bar represents 1μm.
(TIF)

S2 Fig Quantitative PCR analysis of angiogenesis-related genes in IκBα shRNA-mediated transient knockdown HONE1 cells. Angiogenic genes include: *IL6*, *IL8*, Total *VEGF*, *VEGF165*, *VEGF189*, *PDGFB*, and *MCP1*. *GAPDH* was used as an internal control. IκBα-shRNA HONE1 cells displayed an overall increase in angiogenic markers, with significantly higher expression of *IL6* and *IL8* cytokines compared with scramble control cells.
(TIF)

S3 Fig IκBα shRNA-mediated transient knockdown HONE1 cells undergo epithelial-mesenchymal transition (EMT). **(A)** Quantitative PCR analysis of EMT-related genes Fold-differences of the gene expression were compared to the scramble control. EMT genes include: *E-cadherin*, *α-Catenin*, *β-catenin*, *N-cadherin*, *Slug*, *Snail*, *Twist*, *Vimentin*, *SM-Actin*, and *Sox9*. *GAPDH* was used as an internal control. IκBα-shRNA HONE1 cells down-regulated the expression of epithelial markers (*E-cadherin*, *α-Catenin* and *β-catenin*), while inducing the expression of mesenchymal markers (*N-cadherin*, *Slug*, *Snail*, *Twist*, *Vimentin*, *SM-Actin*, and *Sox9*). **(B)** HONE1 brightfield microscopic images of scramble control and IκBa-shRNA cells at 40x magnification. Scale bar represents 10μm.
(TIF)

S1 Table shRNA oligonucleotide sequences. (PDF)

S2 Table Primary antibodies used in the study. (PDF)

S3 Table Gene-specific primers used in the study. (PDF)
