# Supplementary figures and images for: NF-κB p65 Subunit Is Modulated by Latent Transforming Growth Factor-β Binding Protein 2 (LTBP2) in Nasopharyngeal Carcinoma HONE1 and HK1 Cells
Source: PLoS One. 2015 May 14;10(5):e0127239. doi: 10.1371/journal.pone.0127239 (PMC4431814; doi:10.1371/journal.pone.0127239)

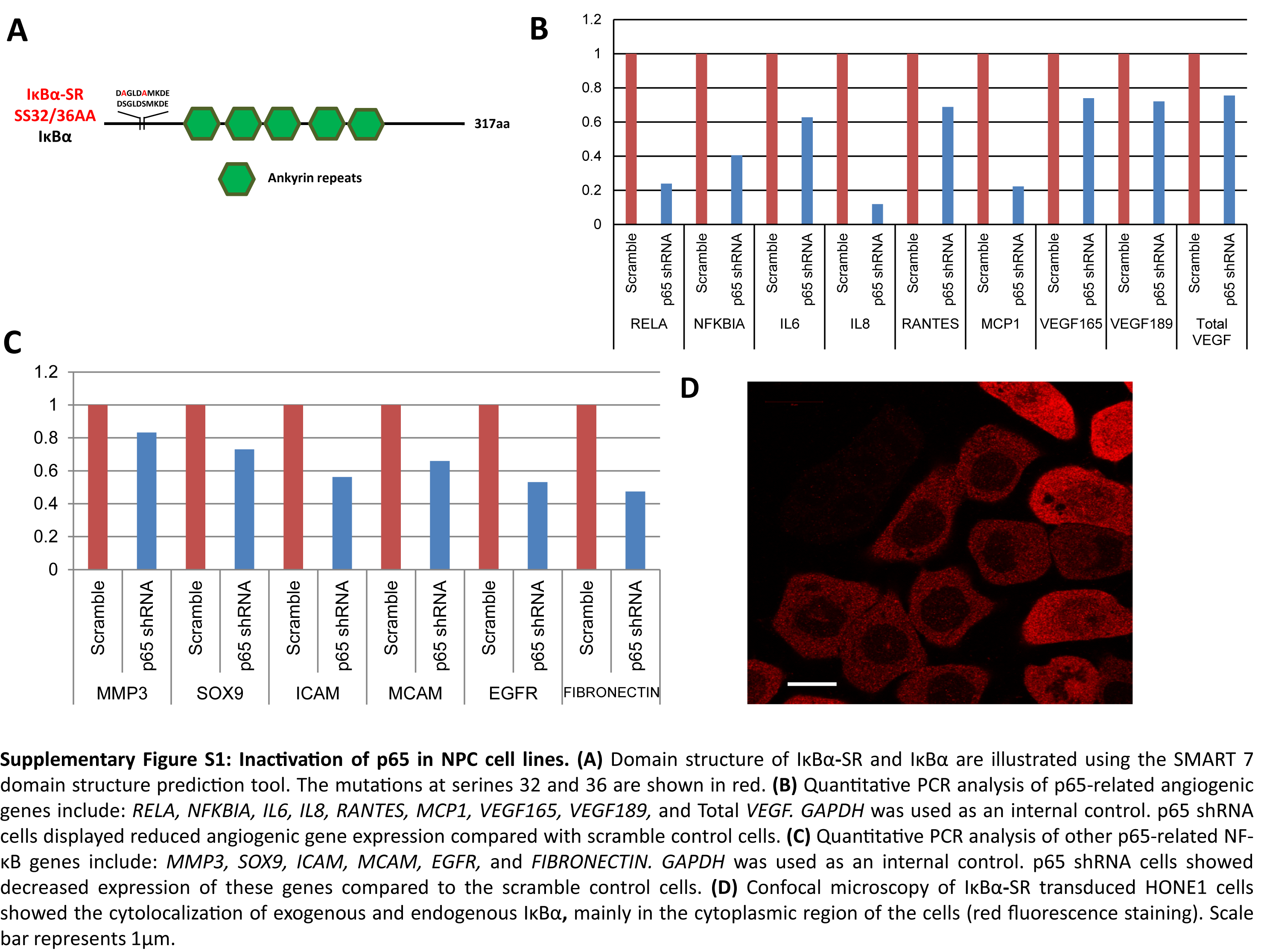

Supplement: S1 Fig — (A) Domain structure of IκBα-SR and IκBα are illustrated using the SMART 7 domain structure prediction tool. The mutations at serines 32 and 36 are shown in red (B) Real-time qPCR analysis of p65-related angiogenic genes include: RELA, NFKBIA, IL6, IL8, RANTES, MCP1, VEGF165, VEGF189, and Total VEGF. GAPDH was used as an internal control. p65 shRNA cells displayed reduced angiogenic gene expression compared with scramble control cells. (C) Quantitative PCR analysis of other p65-related NF-κB genes include: MMP3, SOX9, ICAM, MCAM, EGFR, and FN1. GAPDH was used as an internal control. p65 shRNA cells showed decreased expression of these genes compared to the scramble control cells. (D) Confocal microscopy of IκBα-SR transduced HONE1 cells showed the cytolocalization of exogenous and endogenous IκBα, mainly in the cytoplasmic region of the cells (red fluorescence staining). Scale bar represents 1μm. (TIF) [file pone.0127239.s001.tif]

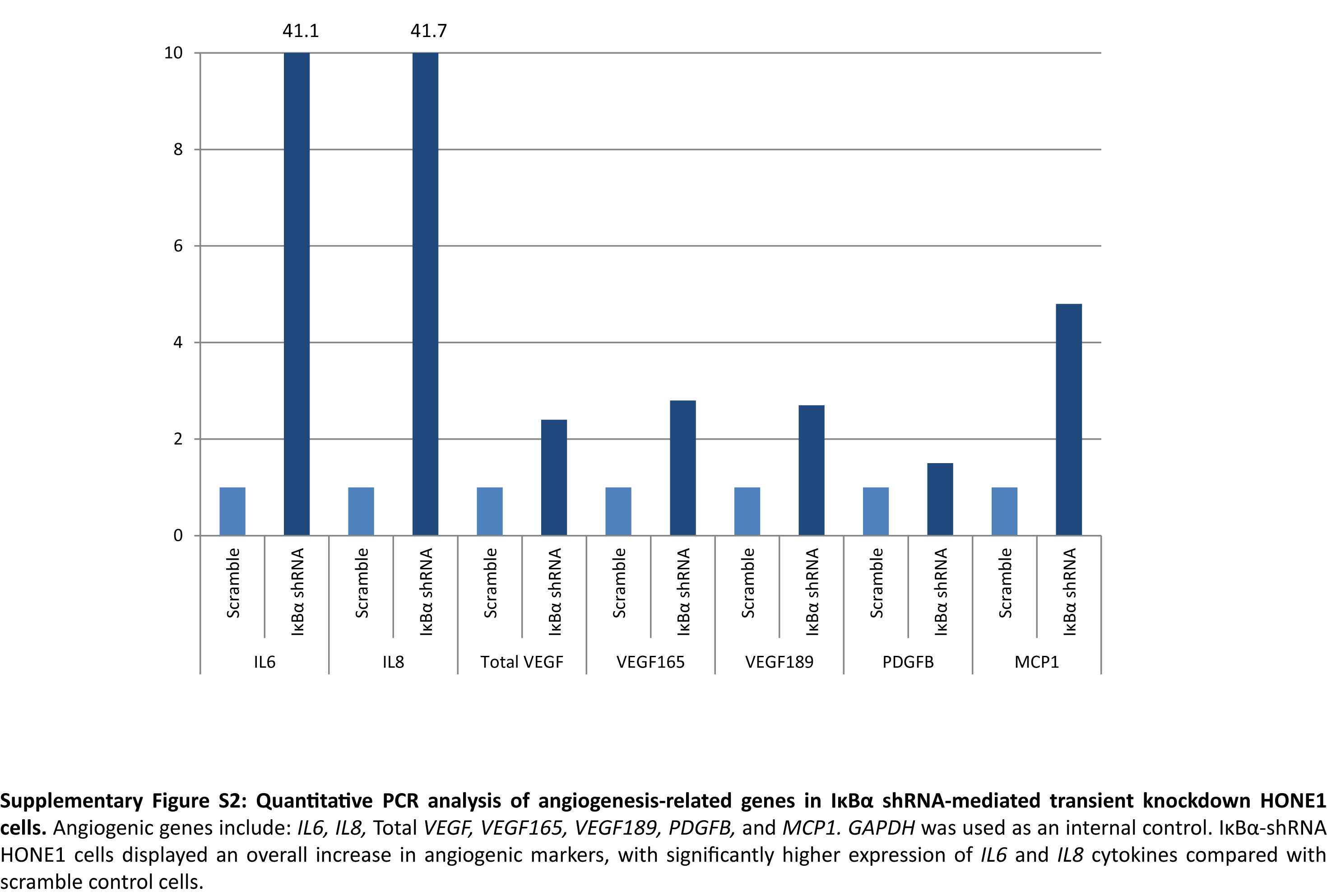

Supplement: S2 Fig — Angiogenic genes include: IL6, IL8, Total VEGF, VEGF165, VEGF189, PDGFB, and MCP1. GAPDH was used as an internal control. IκBα-shRNA HONE1 cells displayed an overall increase in angiogenic markers, with significantly higher expression of IL6 and IL8 cytokines compared with scramble control cells. (TIF) [file pone.0127239.s002.tif]

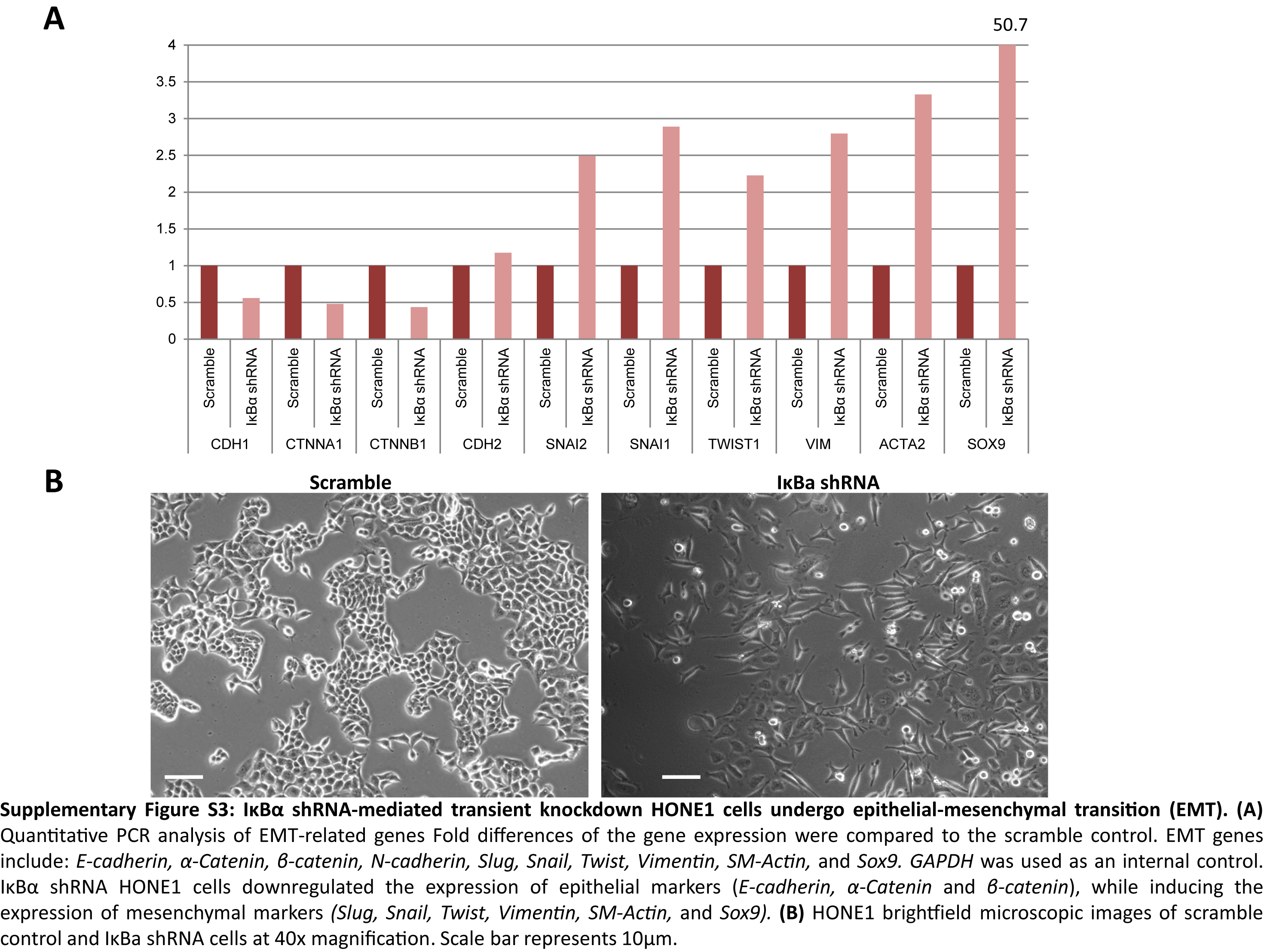

Supplement: S3 Fig — (A) Quantitative PCR analysis of EMT-related genes Fold-differences of the gene expression were compared to the scramble control. EMT genes include: E-cadherin, α-Catenin, β-catenin, N-cadherin, Slug, Snail, Twist, Vimentin, SM-Actin, and Sox9. GAPDH was used as an internal control. IκBα-shRNA HONE1 cells down-regulated the expression of epithelial markers (E-cadherin, α-Catenin and β-catenin), while inducing the expression of mesenchymal markers (N-cadherin, Slug, Snail, Twist, Vimentin, SM-Actin, and Sox9). (B) HONE1 brightfield microscopic images of scramble control and IκBa-shRNA cells at 40x magnification. Scale bar represents 10μm. (TIF) [file pone.0127239.s003.tif]
