# Supplementary material for: NF-κB p65 Subunit Is Modulated by Latent Transforming Growth Factor-β Binding Protein 2 (LTBP2) in Nasopharyngeal Carcinoma HONE1 and HK1 Cells
Source: PLoS One. 2015 May 14;10(5):e0127239. doi: 10.1371/journal.pone.0127239 (PMC4431814; doi:10.1371/journal.pone.0127239)
Supplement: S1 Table — (PDF) [file pone.0127239.s004.pdf]

shRNA	TRC clone number	Region	Target sequence (5' to 3')
p65 sh	TRCN0000014684	CDS	CGGATTGAGGAGAAACGTAAA
IκBα sh	TRCN0000004540	CDS	GCAGCAGACTCCACTCCACTT

Supplementary Table S1: shRNA oligonucleotide sequences
