# Supplementary material for: NF-κB p65 Subunit Is Modulated by Latent Transforming Growth Factor-β Binding Protein 2 (LTBP2) in Nasopharyngeal Carcinoma HONE1 and HK1 Cells
Source: PLoS One. 2015 May 14;10(5):e0127239. doi: 10.1371/journal.pone.0127239 (PMC4431814; doi:10.1371/journal.pone.0127239)
Supplement: S2 Table — (PDF) [file pone.0127239.s005.pdf]

Antibody name	Source	Catalog number	Host	Dilution	Size (kDa)
p-p65 (Ser536)	Cell Signaling Technology Inc.	3033	Rabbit	1:1000	65
p65	Cell Signaling Technology Inc.	8242	Rabbit	1:1000	65
p84		GTX70220	Mouse	1:2000	84
IκBα	Cell Signaling Technology Inc.	4814	Mouse	1:500	40
p-IκBα (Ser32/36)	Cell Signaling Technology Inc.	5210	Rabbit	1:1000	40
IKKα	Cell Signaling Technology Inc.	2682	Rabbit	1:1000	85
IKKβ	Cell Signaling Technology Inc.	8943	Rabbit	1:1000	87
p-IKKα/β (Ser176/180)	Cell Signaling Technology Inc.	2697	Rabbit	1:1000	85-87
LTBP2	Provided by Dr Marko Hyytiainen	-	Rabbit	1:1000	240
