# Supplementary material for: NF-κB p65 Subunit Is Modulated by Latent Transforming Growth Factor-β Binding Protein 2 (LTBP2) in Nasopharyngeal Carcinoma HONE1 and HK1 Cells
Source: PLoS One. 2015 May 14;10(5):e0127239. doi: 10.1371/journal.pone.0127239 (PMC4431814; doi:10.1371/journal.pone.0127239)
Supplement: S3 Table — (PDF) [file pone.0127239.s006.pdf]

Primer name	Sequencing (5' to 3')	Product size (bp)	Anneal temp (°C)
IL6	GCAAAGAGGCACTGGCAGAA	93	60
	GGCAAGTCTCCTCATTGAATCC		
IL8	GGCCGTGGCTCTCTTGGCAG	242	60
	GCACCCAGTTTTCCTTGGGGTCC		
VEGF165	TGTGAATGCAGACCAAAGA	74	60
	TGCTTTCTCCGCTCTGAGC		
VEGF189	CGCAAGAAATCCCGGTATAAGT	65	60
	TGCTTTCTCCGCTCTGAGC		
Total VEGF	ACTGCCATCCAATCGAGACC	76	60
	GATGGCTTGAAGATGTACTCGATCT		
ANG	TGTCCTGCCCGTTTCTGCGG	191	60
	CCGGCCCTGTGGTTTGGCAT		
PDGFB	ATCGCTGCTGGGCGCTCTTC	148	60
	TCTCCGTGCAGCAGGCGTTG		
RANTES	GGCAGCCCTCGCTGTCATCC	114	60
	GGCAGTGGGCGGGCAATGTA		
RELA	TCATGAAGAAGAGTCCTTTCAGC	128	60
	GGATGACGTAAAGGGATAGGG		
NFKBIA	TCCTCAACTTCCAGAACAACC	114	60
	CTCGGAGCTCAGGATCACA		
LTBP2	CTGAACACTGTGAACGGACAG	65	60
	CAGCAGTCCTCCTGGGTAGT		
TWIST	GGAGTCCGCAGTCTTACGAG	201	60
	TCTGGAGGACCTGGTAGAGG		
MMP3	CAAAACATATTTCTTTGTAGAGGACAA	91	60
	TTCAGCTATTTGCTTGGGAAA		
SOX9	AGCCCCAGCCACTACAGCGA	104	60
	CGGGTGATGGGCGGGTAGGA		
ICAM	AACCTCAGCCTCGCTATGG	126	60
	GATGACTTTTGAGGGGGACA		
EGFR	CTATGTGCAGAGGAATTATGATCTTT	76	60
	GAGGGCAATGAGGACATAACC		
FN	CAGTGGGAGACCTCGAGAAG	168	60
	TCCCTCGGAACATCAGAAAC		
GAPDH	GAAGGTGAAGGTGGAGTC	220	60
	GAAGATGGTGATGGGATTTC		

Supplementary Table S3: Gene-specific primers used in the study
